# The Diagnosis of Diseases of the Heart and Thoracic Aorta

**Published:** 1893-06

**Authors:** 


### Reviews of Books.

*The Diagnosis of Diseases of the Heart and Thoracic Aorta.* By A. ERNEST SANSOM, M.D., F.R.C.P. Lond. Pp. xxiii., 567. London: CHARLES GRIFFIN AND COMPANY, LIMITED. 1892.

By diagnosis should be understood a knowledge, as complete as possible, of all the morbid conditions. The discovery of the name of a disease or morbid condition is not the making of a diagnosis. There are many difficulties yet to be surmounted, and light from any trustworthy source should be welcome.

Dr. Sansom's earnest desire is to inculcate precision—precision of observation and precision of record. His method of pleximetric percussion is a great improvement on the ordinary methods, and his graphic records are equally so. The pleximeter consists of a single piece of vulcanite, a larger plate below and a smaller one above, with a connecting vertical column. The outlines obtained by percussion may be drawn upon the chest-wall of the patient; these are again outlined by an oiled brush, and then transferred from the chest-wall to a sheet of copying paper, on which the oil outline is coloured in by the dermatographic pencils. It is obvious that this plan of recording is one that attains the maximum of precision. Many of these graphic records from a variety of typical cases are bound up life-size in the volume, and they are certainly in every sense worthy of the designation "graphic." True it may be that the heart during life is constantly undergoing changes in size and form, and, further, that its condition is much disguised by various pathological states of the lungs; but, nevertheless, much can be learned by accurate methods of percussion, and important inferences drawn therefrom. Further, the author endeavours to show that just as in physiology the sphygmograph and cardiograph have contributed in a very important degree to advance our knowledge of the processes of the circulation, so these instruments may be of value in the more intricate problems of cardiac pathology. The contents of this book show that the hard-working observer may obtain ample evidence which is not open to him who runs, and that the problems of the circulation can only be elucidated by painstaking and persevering care. The utility of precise records taken by pleximeter, sphygmograph, and cardiograph is abundantly shown, and that "the way of diagnosis is hard" is amply proved.

The book is a great advance on any previous publication:

it contains the best work of one of our best observers, and it is presented to us in a most attractive form—easy to read and tempting to study. A brief review could not do even bare justice to such a book: it requires to be closely and perseveringly studied. There are some books which have cost the authors a lifetime of work, and require almost a corresponding time for digestion and assimilation by the reader; but this book is not quite so exacting, inasmuch as Dr. Sansom gives his arguments and conclusions clearly and concisely, without any excess of statistics or verbosity.

We may quote one paragraph on the much-discussed question of the pathogenesis of the murmurs heard over the pulmonary artery and over the aorta. He writes (p. 285):

"To come to first principles, these are due to vibrations communicated to the area of the thorax superficial to these vessels, the vibrations being first communicated to the portions of the vessels immediately above the valves. The difficult point is, where are these vibrations initiated? It is unlikely that any vaso-motor affection of the great arteries themselves can be invoked as an explanation. The great arterial pouches must vibrate only through communicated vibrations. Their coats are probably not lax, for the tension in both of them in the majority of cases is greater, and not less than the normal. The cause reverts, therefore, with great probability to the conus of the ventricle immediately below the valves. It is this part of the ventricle that has to bear the greatest strain during the ventricular systole, as it is immediately below the weight to be overcome; in the case of the right ventricle, the pulmonic valves, closed by reason of the tension in the pulmonary artery and its branches. The ventricle, weakened by impaired nerve-force, toils to overcome it, and that the obstruction is greater than the normal the accentuated pulmonic second sound testifies. Analogy tells us that the effect of overstrain on voluntary muscle is tremor. So, I take it, fibrillar tremor can be initiated at the overstrained portion of the right ventricle, the conus just below the valves. It seems quite possible that the valves themselves may vibrate in the current. I have noticed in the case of hardened specimens of hearts in firm systolic contraction how pronounced are the muscular bundles carried far into the base of the pulmonic valves. It seemed to me that these might have their function in keeping the semilunes in their due place during diastole.

"The aortic murmur in anæmia and in neuroses is to be explained in an analogous manner. The right ventricle and its conus are more superficial, its muscular walls are thinner, and, in the condition named, it has to contend against a relatively greater obstruction: therefore, the murmurs over its site are more frequent. The apex-murmur due to changes in the left ventricle is, however, by no means infrequent, and its mechanism we have already considered. The aortic murmur is more rare, but by no means very unusual: it is due to an impairment of the tonus in the muscle of the conus of the left ventricle."

